# Supplementary material for: Excessive copper impairs intrahepatocyte trafficking and secretion of selenoprotein P
Source: Nat Commun. 2023 Jun 13;14:3479. doi: 10.1038/s41467-023-39245-3 (PMC10264388; doi:10.1038/s41467-023-39245-3)
Supplement: Supplementary file 1 — Supplementary Information [file 41467_2023_39245_MOESM1_ESM.pdf]

Supplementary Information File

Excessive copper impairs intrahepatocyte trafficking and secretion of selenoprotein P

Maria Schwarz[1,2], Caroline E. Meyer[1,2], Alina Löser[1,2], Kristina Lossow[1,2], Julian Hackler[2,3], Christiane Ott[2,4], Susanne Jäger[2,5], Isabelle Mohr[6], Ella A. Eklund[7,8], Angana A.H. Patel[7,8], Nadia Gul[7,8], Samantha Alvarez[7,8], Ilayda Altinonder[7,8], Clotilde Wiel[7,8], Maria Maares[2,9], Hajo Haase[2,9], Anetta Härtlova[8,10,11], Tilman Grune[2,4], Matthias B. Schulze[2,5,12], Tanja Schwerdtle[2,12,13], Uta Merle[6], Hans Zischka[14,15], Volkan I. Sayin[7,8], Lutz Schomburg[2,3], Anna P. Kipp[1,2]*

[1]Department of Nutritional Physiology, Institute of Nutritional Sciences, Friedrich Schiller University Jena, Dornburger Str. 24, 07743 Jena, Germany

[2]TraceAge-DFG Research Unit on Interactions of Essential Trace Elements in Healthy and Diseased Elderly, Potsdam-Berlin-Jena-Wuppertal, Germany

[3]Institute for Experimental Endocrinology, Charité - University Medical School Berlin, Hessische Straße 3-4, 10115 Berlin, Germany

[4]Department of Molecular Toxicology, German Institute of Human Nutrition Potsdam-Rehbrücke, Arthur-Scheunert-Allee 114-116, 14558 Nuthetal, Germany

[5]Department of Molecular Epidemiology, German Institute of Human Nutrition Potsdam-Rehbrücke, Arthur-Scheunert-Allee 114-116, 14558 Nuthetal, Germany

[6]Department of Internal Medicine IV, University Hospital Heidelberg, Im Neuenheimer Feld 672, 69120 Heidelberg, Germany

[7]Institute of Clinical Sciences, Department of Surgery, Sahlgrenska Center for Cancer Research, University of Gothenburg, Blå stråket 5, 41345 Gothenburg, Sweden

[8]Wallenberg Centre for Molecular and Translational Medicine, University of Gothenburg, 41345 Gothenburg, Sweden

[9]Department of Food Chemistry and Toxicology, Technical University Berlin, Gustav-Meyer-Allee 25, 13355 Berlin, Germany

[10]Institute of Biomedicine, Department of Microbiology and Immunology, University of Gothenburg, 41345 Gothenburg, Sweden

[11]The Institute of Medical Microbiology and Hygiene, University Medical Centre Freiburg, Freiburg, Germany

[12]Institute of Nutritional Science, University of Potsdam, Arthur-Scheunert-Allee 114-116, 14558 Nuthetal, Germany

[13]German Federal Institute for Risk Assessment (BfR), Max-Dohrn-Str. 8-10, 10589 Berlin, Germany

[14]Institute of Toxicology and Environmental Hygiene, Technical University Munich, School of Medicine, Biedersteinerstraße 29 80802 Munich, Germany

[15]Institute of Molecular Toxicology and Pharmacology, Helmholtz Center Munich, German Research Center for Environmental Health, Ingolstädter Landstraße 1, 85764 Neuherberg, Germany

These authors contributed equally: Maria Schwarz, Caroline E. Meyer

*anna.kipp@uni-jena.de

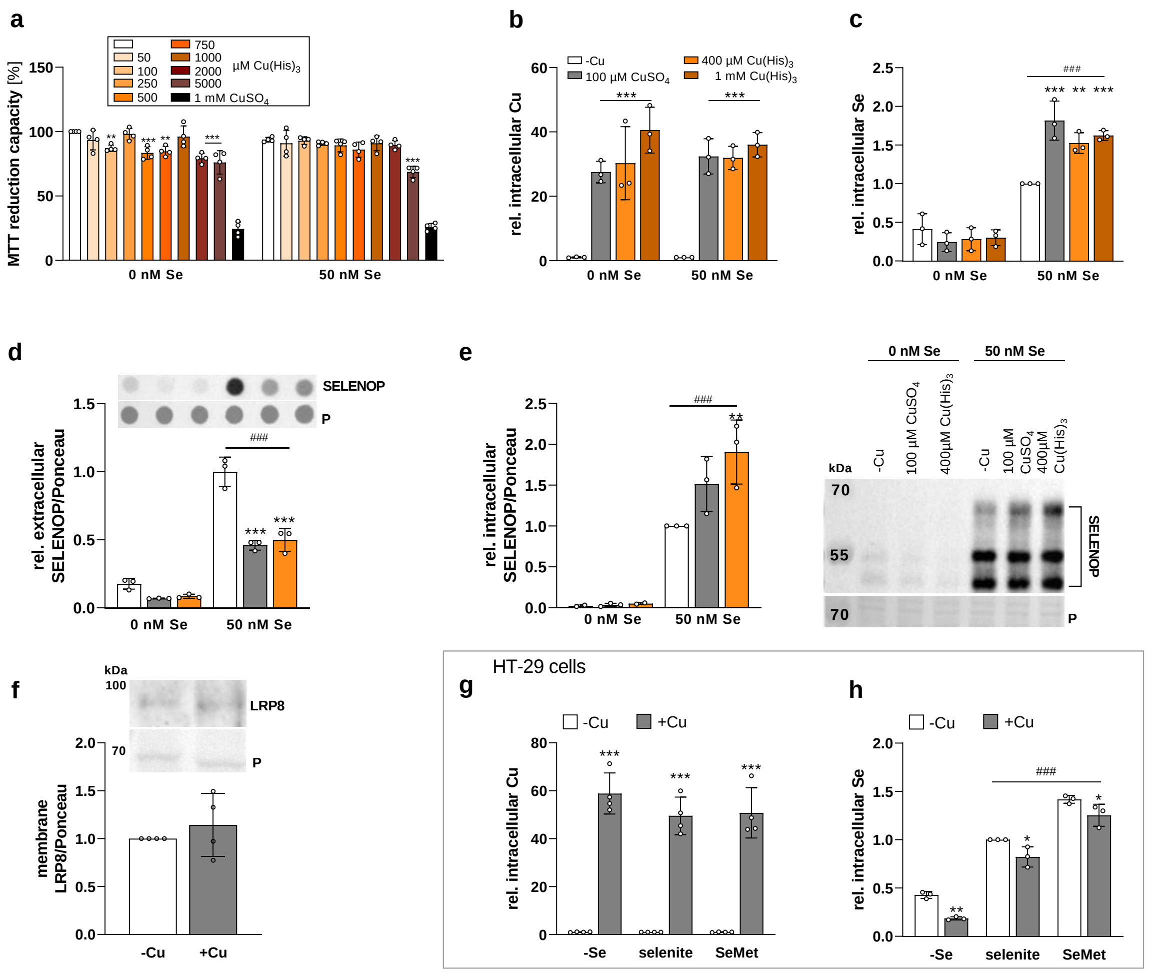

Supplementary Figure 1. Copper histidine also interferes with selenium homeostasis in HepG2 cells. (**a**) MTT reduction capacity of HepG2 cells treated with increasing concentrations of Cu(His)$_3$ or 1 mM $CuSO_4$ in combination with or without 50 nM selenite for 72 h measured photometrically with untreated cells set as 100 % (n=4). Intracellular Cu (**b**) and Se (**c**) concentrations of HepG2 cells cultured without Cu, 100 µM $CuSO_4$, 400 µM Cu(His)$_3$ or 1 mM Cu(His)$_3$ in combination with or without 50 nM selenite for 72 h measured using TXRF with 1 mg/L yttrium as standard element for 1000 s. The trace element concentrations were normalized to protein content (n=3). (**d**) Extracellular SELENOP of HepG2 cells treated as in B determined using dot blot and normalized to Ponceau staining (n=3). (**e**) Intracellular SELENOP levels analyzed using Western Blot, normalized to Ponceau staining with selenite-treated samples set to 1 (n=3). (**f**) Localization of LRP8 in the membrane/ organelle fraction determined by Western Blot and normalized to Ponceau staining of cells treated for 72 h with 50 nM selenite in combination with or without 100 µM $CuSO_4$ (n=4). Intracellular Cu (n=4) (**g**) and Se (n=3) (**h**) concentrations of HT29 cells treated with 0 or 100 µM $CuSO_4$ in combination with or without 50 nM selenite or 200 nM SeMet and determined using TXRF with 1 mg/L Yttrium as standard for 1000 s and normalized to protein content. Data are depicted as mean ± SD. Biological replicates are indicated by individual dots. Statistical analyses were based on

two-way ANOVA with Bonferroni's post-test. *p < 0.05; **p < 0.01; ***p < 0.001 vs. -Cu; ###p < 0.001 vs. -Se or by two-tailed t-test compared to cells without Cu treatment (**f**). Source data are provided as Source Data file.

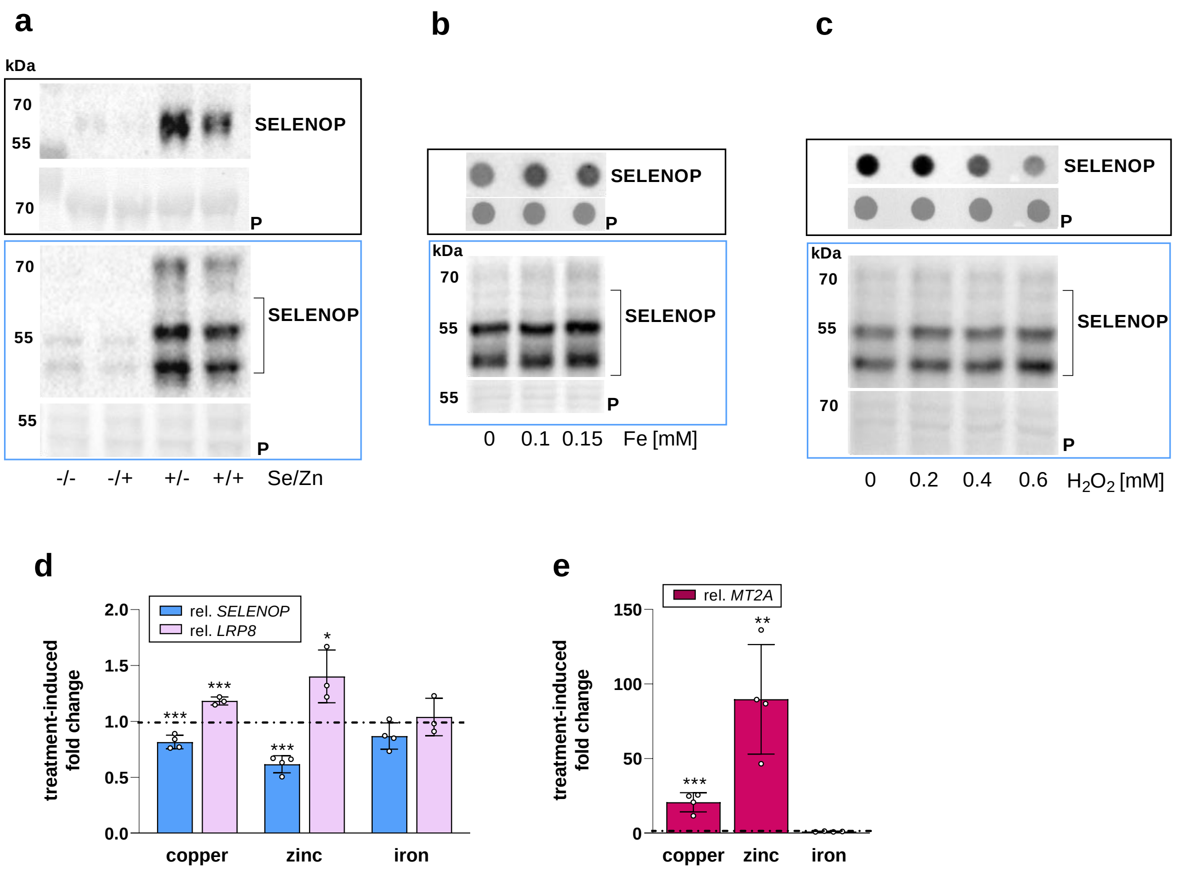

Supplementary figure 2. Effects of zinc, iron, and H_2O_2 on SELENOP expression and distribution. Representative Western Blots for Zn- (**a**), Fe- (**b**), and H_2O_2-induced (**c**) effects on extra- (black boxes) and intracellular (blue boxes) SELENOP. HepG2 cells were treated with 0 or 100 µM $ZnSO_4$ in combination with 0 or 50 nM selenite (**a**) or selenite-cultured cells were treated with 0, 0.1 or 0.15 mM $FeCl_2$ (**b**) or 0, 0.2, 0.4 or 0.6 mM H_2O_2 (**c**). All experiments were repeated independently four times with similar results. Relative mRNA expression of *SELENOP* (n=4), *LRP8* (n=3) (**d**) or *MT2A* (n=4) (**e**) was determined by qPCR after 24 h incubation with 0 or 100 µM $CuSO_4$, 100 µM $ZnSO_4$ or 100 µM $FeCl_2$ and normalized to *RPL13A*. Data are shown as treatment-induced fold change and untreated cells were set as 1 (dotted line). Data are depicted as mean ± SD. Biological replicates are indicated by individual dots. Statistical analyses were based on two-tailed t-test compared to untreated cells (**d-e**). *p < 0.05; **p < 0.01; ***p < 0.001 vs. untreated cells. Source data are provided as Source Data file.

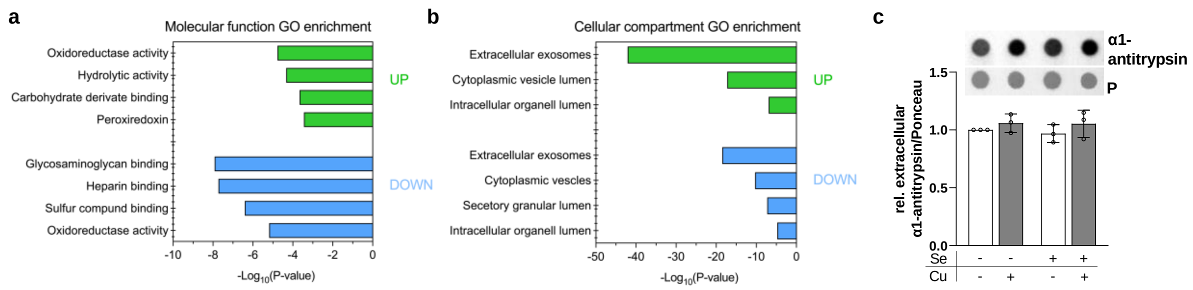

Supplementary Figure 3. Secretome analyses of Cu-treated HepG2 cells. GO enrichment of proteins identified by secretome analysis for 'Molecular function' (**a**) and 'Cellular compartment' (**b**). Analyses were performed in medium samples derived from HepG2 cells treated for 48 h with 50 nM selenite with 0 or 100 µM $CuSO_4$. t-Test-based statistics were applied on normalized and logarithmized protein ratios to extract the significantly regulated proteins. Functional annotation of proteins by Gene Ontology (GO) was done using DAVID Gene Ontology enrichment analysis. (**c**) Extracellular α1-antitrypsin of HepG2 cells treated for 72 h with or without 50 nM selenite in combination with 0 or 100 µM $CuSO_4$ was analyzed using Western Blot, normalized to Ponceau (P) staining, and untreated samples set to 1 (n=3). Data are depicted as mean ± SD. Biological replicates are indicated by individual dots. Statistical analyses were based on two-way ANOVA with Bonferroni's post-test. Source data are provided as Source Data file.

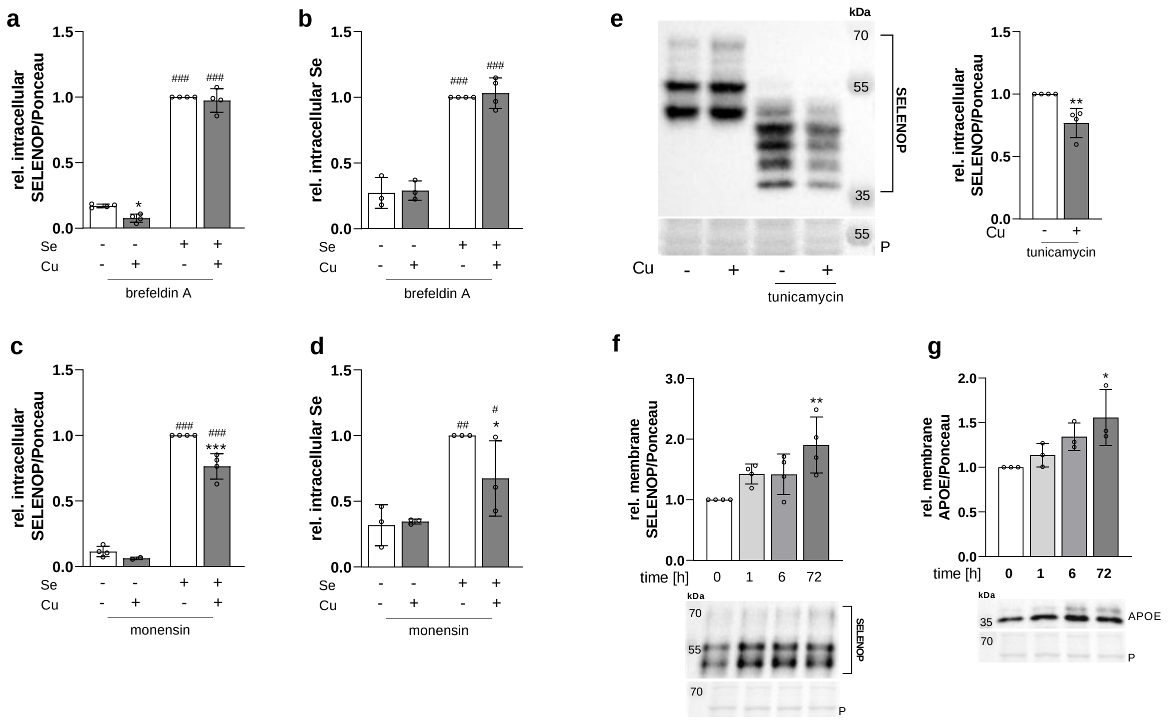

Supplementary Figure 4. Effects of modulated Golgi trafficking on SELENOP. Intra-cellular SELENOP (**a, c, e**) (n=4) and intracellular Se (**b** (n=4)**, d** (n=3)) of HepG2 cells treated with 0 or 100 µM $CuSO_4$ without (-Se) or with 50 nM selenite (+Se) for 72 h. 24 h prior to harvest brefeldin A (**a, b**), monensin (**c, d**), or tunicamycin (**e**) (n=4) were added. Samples were analyzed by Western Blot normalized to Ponceau (P) staining or by TXRF. SELENOP (**f**) (n=4) and APOE (**g**) (n=3) localization in the membrane/ organelle fraction of cells treated with 50 nM selenite in combination with or without 100 µM $CuSO_4$ for indicated time points. Representative images are show for all Western Blots. Data are depicted as mean ± SD. Biological replicates are indicated by individual dots. Statistical analyses were based on two-way ANOVA (**a-d**) or on one-way ANOVA (**f**) with Bonferroni's post-test or two-tailed t-test (**e**). *p < 0.05; **p < 0.01; ***p < 0.001 vs. -Cu; $^{\#}$p < 0.05; $^{\#\#\#}$p < 0.001 vs. -Se. Source data are provided as Source Data file.

Supplementary Table 1. Clinical characterization by serum analyses of the investigated Wilson's disease patients. Data are depicted as mean ± SD. Statistical analyses were based on one-way ANOVA with Bonferroni's post-test. *p<0.05 vs. 'normal CP'; #p<0.05 vs. 'low CP'. LDH = lactate dehydrogenase; GOT = glutamic oxaloacetic transaminase; GPT = glutamic pyruvic transaminase; AP = alkaline phosphatase; GGT = γ-glutamyl transferase; CHE = cholinesterase; CRP = C-reactive protein; n.d. = not detectable

group	sex	age	LDH [U/L]	GOT [U/L]	GPT [U/L]	AP [U/L]	GGT [U/L]	CHE [kU/L]	bilirubin [mg/dL]		albumin [g/L]	CRP [mg/L]
									total	direct		
normal CP	male	30-35	213	29	51	88	39	5.87	0.8	0.20	43.1	2.00
	male	20-25	158	34	32	100	117	4.22	0.6	0.16	45.4	2.00
	male	25-30	148	71	62	79	102	4.25	0.5	0.16	38.1	2.00
	male	25-30	225	48	105	133	45	5.07	1.0	-	45.4	2.00
	female	20-25	219	32	38	127	20	9.98	0.6	0.20	46.9	n. d.
	male	30-35	-	29	48	63	24	11.03	1.4	0.28	51.0	n. d.
	female	20-25	167	30	32	87	36	1.92	0.8	0.20	44.0	n. d.
	female	25-30	201	70	170	80	75	6.23	1.0	0.30	44.0	n. d.
**mean ± SD**		**26±4**	**190±31.7**	**42.9±18.1**	**67.3±47.8**	**94.6±24.2**	**57.3±36.5**	**6.07±3.05**	**0.84±0.29**	**0.21±0.06**	**44.7±3.64**	**2.00±0.00**
low CP	male	15-20	175	43	45	118	67	3.74	0.7	0.30	36.8	2.00
	male	35-40	176	56	115	66	43	6.53	0.6	0.20	42.0	2.00
	male	25-30	177	41	69	116	87	3.94	1.0	0.40	40.0	n. d.
	male	25-30	165	17	14	74	19	5.84	0.8	0.30	45.8	n. d.
	male	35-40	171	23	42	115	107	4.47	0.3	0.10	44.5	5.60
	male	15-20	183	19	19	182	17	10.47	0.4	0.10	47.9	n. d.
	male	25-30	226	80	249	94	143	10.65	0.6	0.20	53.0	n. d.
	male	20-25	191	67	122	110	49	6.77	0.6	0.20	47.5	n. d.
	female	20-25	164	17	20	107	20	4.92	0.8	0.25	47.6	2.00
	female	15-20	210	25	26	78	17	6.92	0.7	0.20	48.3	2.00
	female	25-30	162	13	16	131	14	7.46	0.7	0.18	44.2	2.00
**mean ± SD**		**25±7**	**182±20.1**	**36.5±22.8**	**67.0±71.5**	**108±32.0**	**53.0±43.4**	**6.52±2.35**	**0.65±0.19**	**0.22±0.09**	**45.2±4.45**	**2.60±1.47**
diseased	male	25-30	555	151	108	259	261	1.13	2.40	1.70	32.2	10.1
	female	20-25	275	153	170	117	80	1.05	2.10	-	36.6	17.5
	male	30-35	313	61	114	156	224	2.49	1.00	-	28.7	n. d.
	male	20-25	255	53	18	143	76	1.48	4.40	1.30	43.1	8.50
**mean ± SD**		**26±4**	**350±139 * #**	**105±55.0 * #**	**103±62.9**	**169±62.3**	**160±96.2 * #**	**1.54±0.66 * #**	**2.48±1.42 * #**	**1.5±0.28 * #**	**31.7±3.65 * #**	**12.0±4.80 * #**

Extended Western Blots

S1E

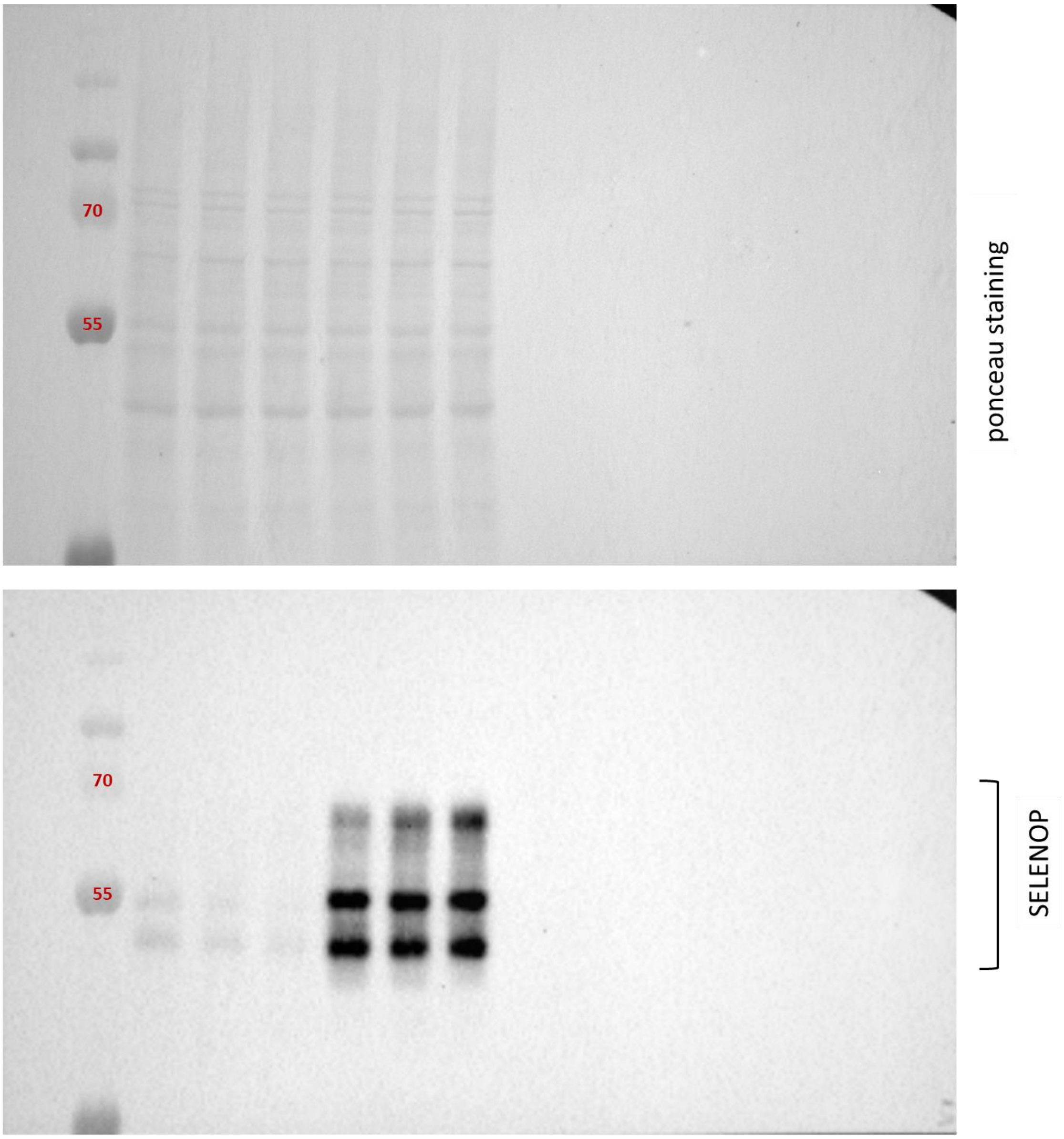

Se	-			+		
Cu	0	CuS	CuH	0	CuS	CuH

CuS: 100 µM $CuSO_4$
CuH: 400 µM $Cu(His)_3$

S1F

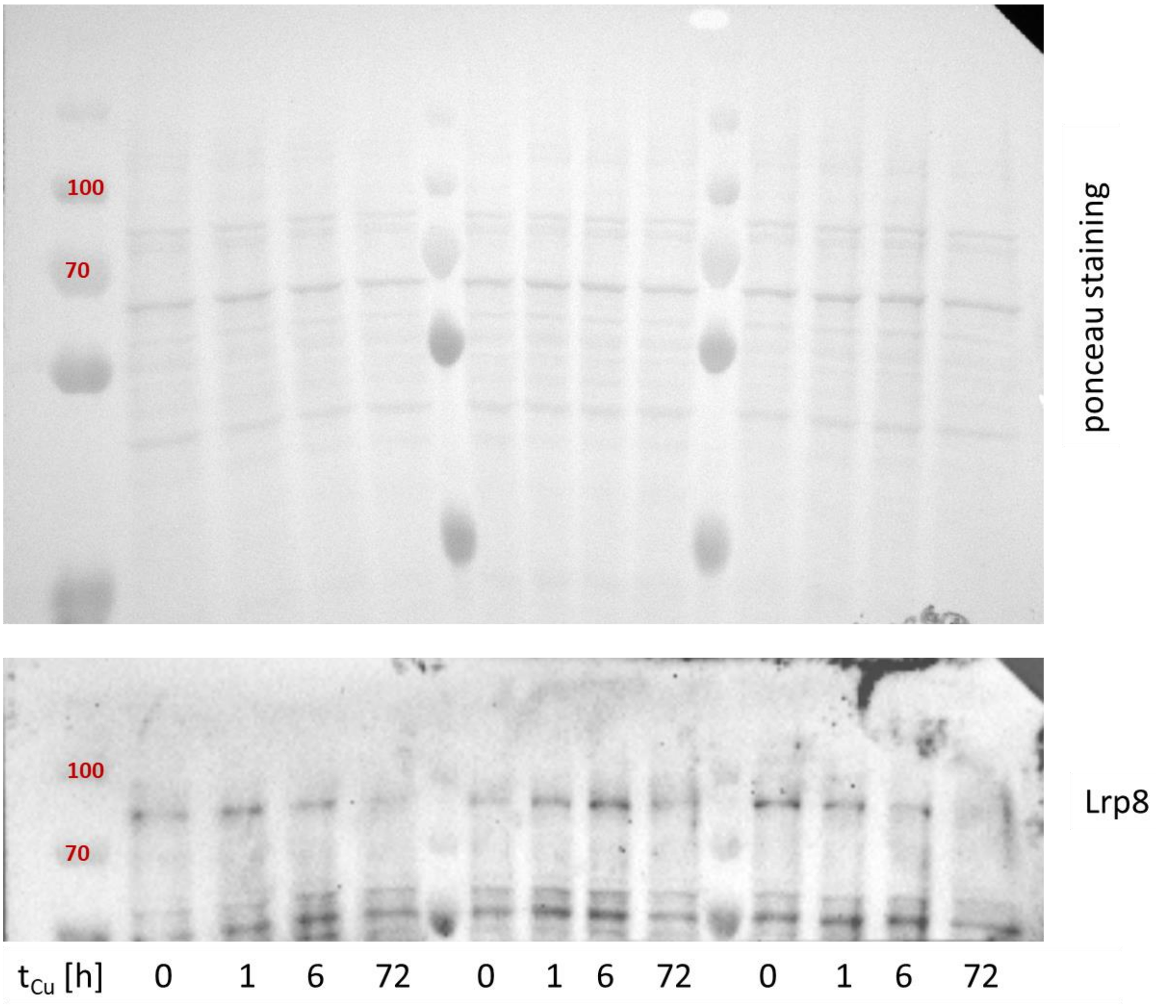

S2A

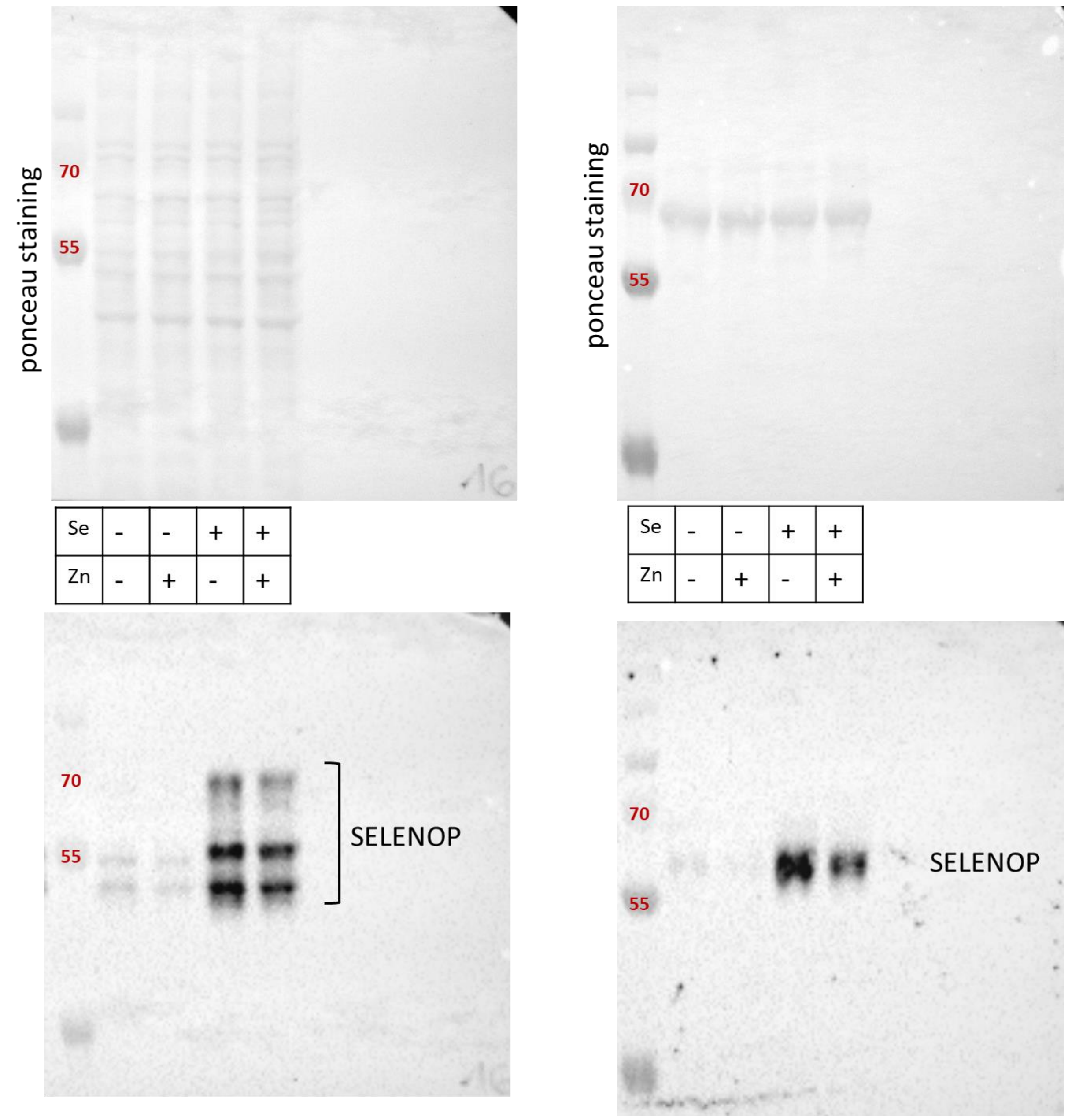
ponceau staining
70
55
Se - - + +
Zn - + - +
70
55
SELENOP
ponceau staining
70
55
Se - - + +
Zn - + - +
70
55
SELENOP

S2B

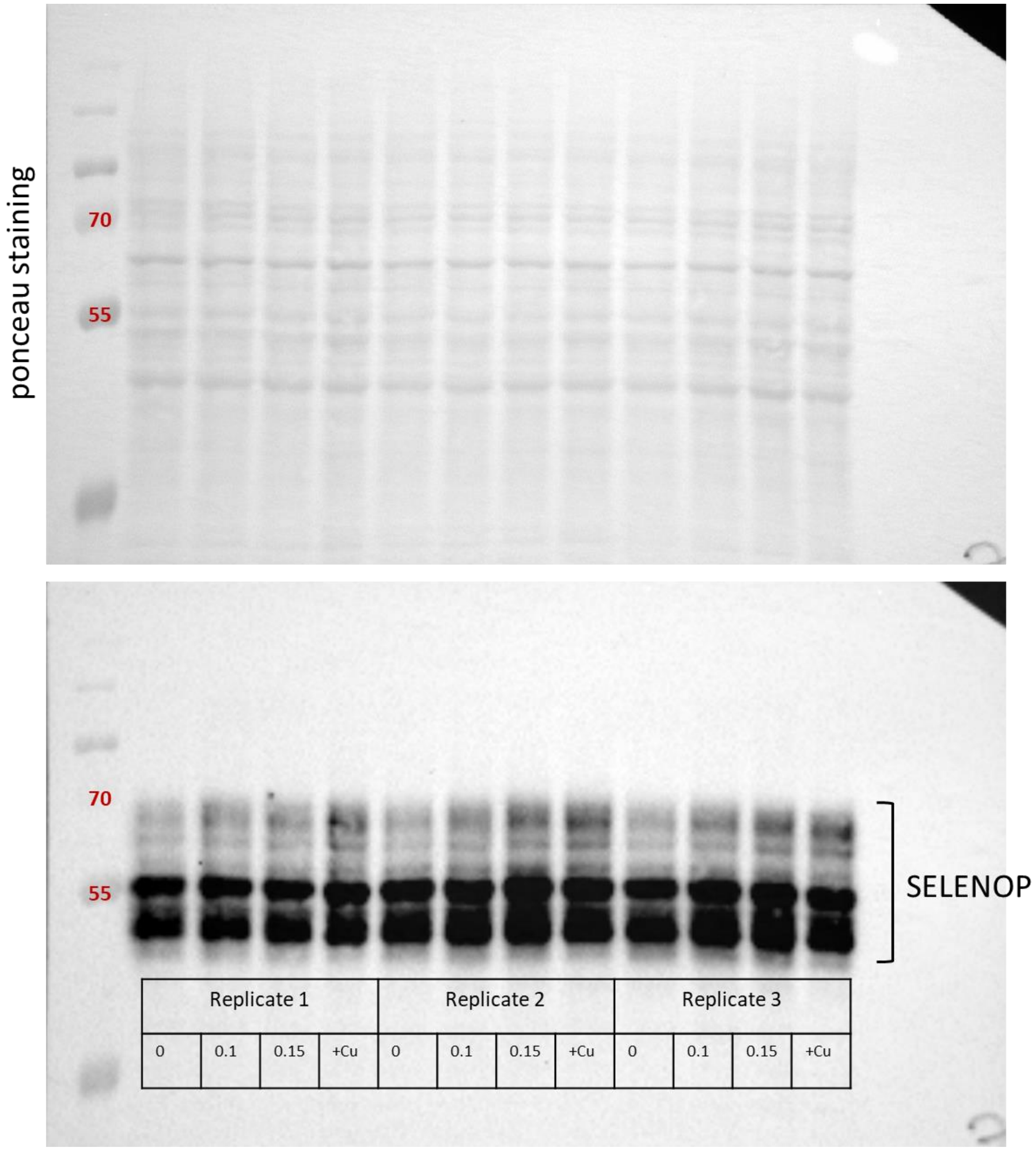
ponceau staining
70
55
70
55
SELENOP
Replicate 1
Replicate 2
Replicate 3
0
0.1
0.15
+Cu
0
0.1
0.15
+Cu
0
0.1
0.15
+Cu

S2C

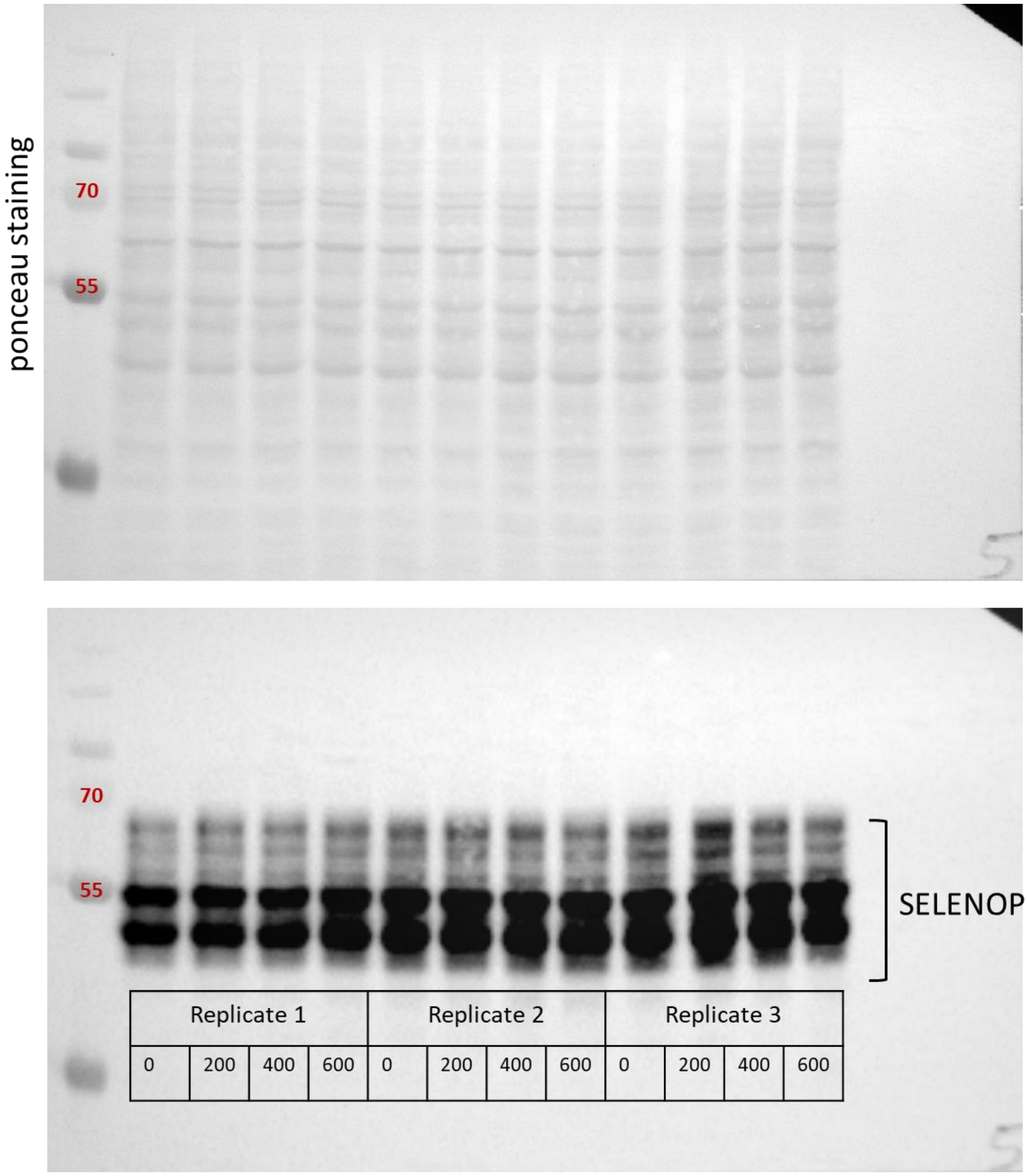
ponceau staining
70
55
70
55
SELENOP
Replicate 1
Replicate 2
Replicate 3
0
200
400
600
0
200
400
600
0
200
400
600

S4E

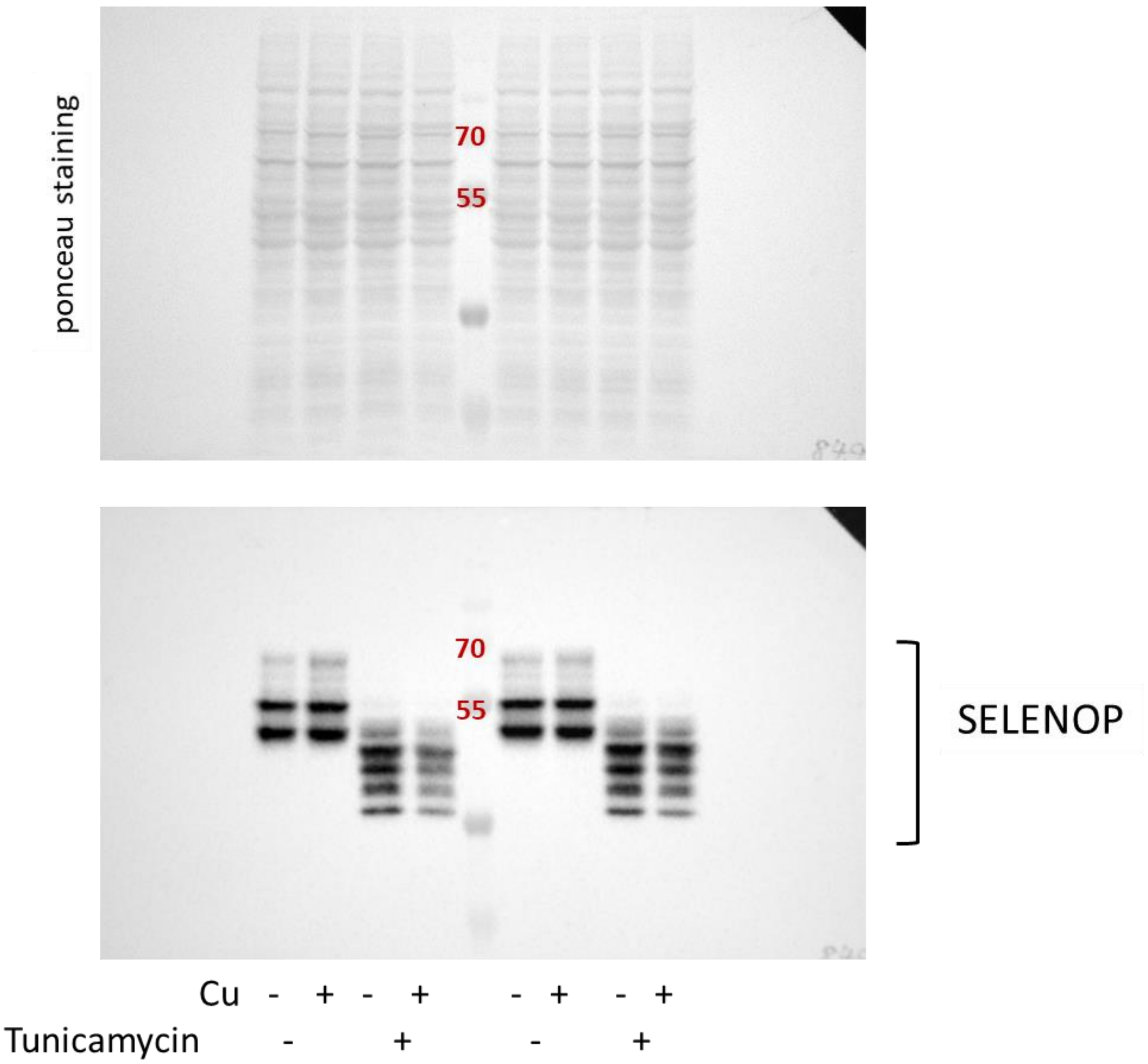

S4F

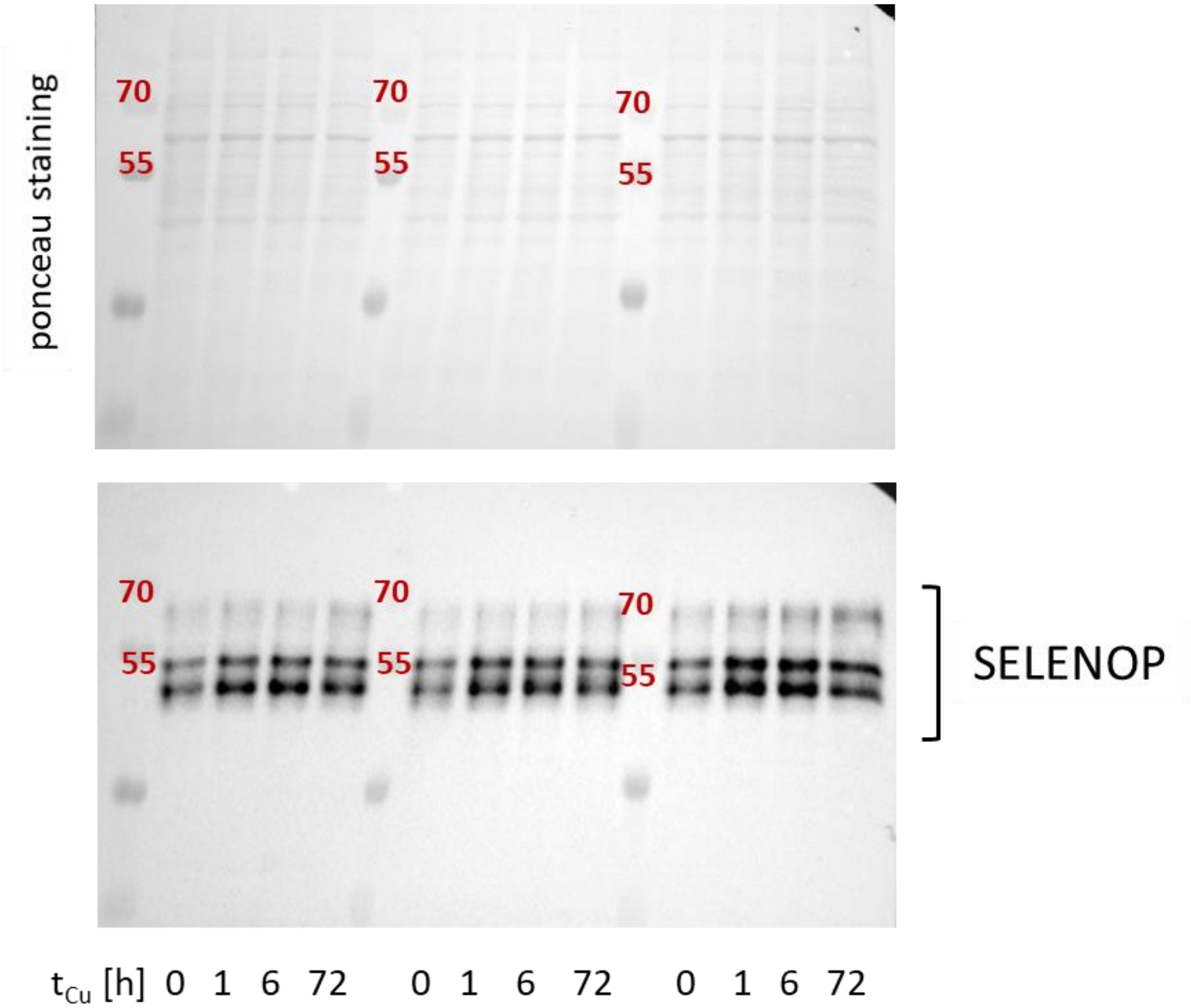
ponceau staining
70
55
70
55
70
55
70
55
70
55
70
55
SELENOP
t_Cu [h] 0 1 6 72 0 1 6 72 0 1 6 72

S4G

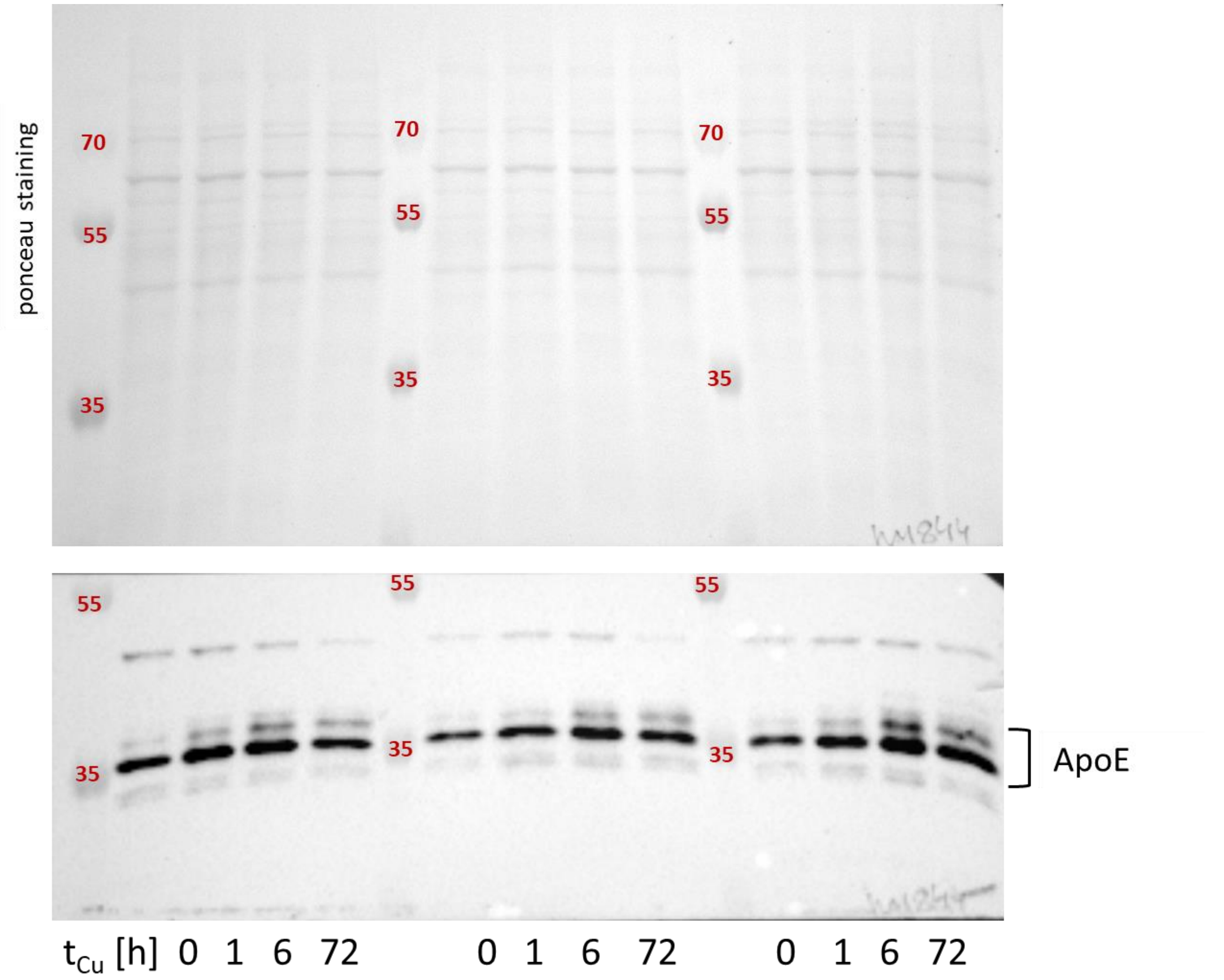

ponceau staining
70
55
35
70
55
35
70
55
35
55
35
55
35
55
35
ApoE
t_{Cu} [h] 0 1 6 72 0 1 6 72 0 1 6 72
