# Supplementary material for: Excessive copper impairs intrahepatocyte trafficking and secretion of selenoprotein P
Source: Nat Commun. 2023 Jun 13;14:3479. doi: 10.1038/s41467-023-39245-3 (PMC10264388; doi:10.1038/s41467-023-39245-3)
Supplement: Supplementary file 3 — Description of Additional Supplementary Files [file 41467_2023_39245_MOESM3_ESM.pdf]

Description of Additional Supplementary Files

Supplementary Data 1: Secretome proteomics data of copper-stimulated HepG2 cells compared to cells without copper. t-Test-based, two-sided statistics were applied on normalized and logarithmized protein ratios to extract the significantly regulated proteins.
