# Supplementary material for: Excessive copper impairs intrahepatocyte trafficking and secretion of selenoprotein P
Source: Nat Commun. 2023 Jun 13;14:3479. doi: 10.1038/s41467-023-39245-3 (PMC10264388; doi:10.1038/s41467-023-39245-3)
Supplement: Supplementary file 5 — Reporting Summary [file 41467_2023_39245_MOESM5_ESM.pdf]

nature portfolio

Corresponding author(s): Anna Kipp

Last updated by author(s): May 25, 2023

Reporting Summary

Nature Portfolio wishes to improve the reproducibility of the work that we publish. This form provides structure for consistency and transparency in reporting. For further information on Nature Portfolio policies, see our Editorial Policies and the Editorial Policy Checklist.

Statistics

For all statistical analyses, confirm that the following items are present in the figure legend, table legend, main text, or Methods section.

n/a	Confirmed	
☐	☒	The exact sample size (*n*) for each experimental group/condition, given as a discrete number and unit of measurement
☐	☒	A statement on whether measurements were taken from distinct samples or whether the same sample was measured repeatedly
☐	☒	The statistical test(s) used AND whether they are one- or two-sided<br>*Only common tests should be described solely by name; describe more complex techniques in the Methods section.*
☐	☒	A description of all covariates tested
☐	☒	A description of any assumptions or corrections, such as tests of normality and adjustment for multiple comparisons
☐	☒	A full description of the statistical parameters including central tendency (e.g. means) or other basic estimates (e.g. regression coefficient) AND variation (e.g. standard deviation) or associated estimates of uncertainty (e.g. confidence intervals)
☐	☒	For null hypothesis testing, the test statistic (e.g. *F*, *t*, *r*) with confidence intervals, effect sizes, degrees of freedom and *P* value noted<br>*Give P values as exact values whenever suitable.*
☒	☐	For Bayesian analysis, information on the choice of priors and Markov chain Monte Carlo settings
☐	☒	For hierarchical and complex designs, identification of the appropriate level for tests and full reporting of outcomes
☐	☒	Estimates of effect sizes (e.g. Cohen's *d*, Pearson's *r*), indicating how they were calculated

Our web collection on statistics for biologists contains articles on many of the points above.

Software and code

Policy information about availability of computer code

Data collection	Western and Dot blots were quantified by ChemiDocTM MP Imaging System (Biorad) using Image Lab software version 5.0. Enzyme activity measurements were performed on a microplate reader (Synergy, Biotek) using Gen5 software version 3.11. Trace element concentrations were measured using a total reflection X-ray fluorescence (TXRF) spectrometer (S4 T-Star, Bruker) and TEsprit software version 1.0. The Mx3005P QPCR System (Agilent Technologies, Santa Clara, CA, USA) was used for qPCR analyses. BioRender was used to generate the scheme provided as Fig. 6e and Fig. 7.
Data analysis	GraphPad Prism version 8 was used for statistical analyses. Proteomic datasets were processed by MaxQuant ver. 1.6.10.43.

For manuscripts utilizing custom algorithms or software that are central to the research but not yet described in published literature, software must be made available to editors and reviewers. We strongly encourage code deposition in a community repository (e.g. GitHub). See the Nature Portfolio guidelines for submitting code & software for further information.

Data

Policy information about availability of data

All manuscripts must include a data availability statement. This statement should provide the following information, where applicable:
- Accession codes, unique identifiers, or web links for publicly available datasets
- A description of any restrictions on data availability
- For clinical datasets or third party data, please ensure that the statement adheres to our policy

The mass spectrometry proteomics data have been deposited to the ProteomeXchange Consortium (http://proteomecentral.proteomexchange.org) via the PRIDE partner repository [28] with the dataset identifier PXD036300. Reviewers can access the data through the Username: reviewer_pxd036300@ebi.ac.uk and the Password: 7TZOkvgT. As reference proteome of Homo Sapiens UP000005640 was used.

Human research participants

Policy information about studies involving human research participants and Sex and Gender in Research.

Reporting on sex and gender	Table S1 the information about sex was based on self-reporting.
Population characteristics	Population characteristics have been described in Table S1.
Recruitment	This is a clinical study. Patients have been recruited in the University Hospital Heidelberg.
Ethics oversight	The study was approved by the Medical Ethics Committee of the University Hospital Heidelberg (Reference no. S-565/2011; DRKS00031527).

Note that full information on the approval of the study protocol must also be provided in the manuscript.

Field-specific reporting

Please select the one below that is the best fit for your research. If you are not sure, read the appropriate sections before making your selection.

☒ Life sciences ☐ Behavioural & social sciences ☐ Ecological, evolutionary & environmental sciences

For a reference copy of the document with all sections, see nature.com/documents/nr-reporting-summary-flat.pdf

Life sciences study design

All studies must disclose on these points even when the disclosure is negative.

Sample size	Sample size determination is based on previous experience to obtain significant results and reproducibility (Schwarz et al., 2020, Redox Biol). The sample size follows common standards employing three or more biological replicates. All sample sizes are listed in each figure legend.
Data exclusions	No data exclusion.
Replication	For cell culture experiments three biological replicates were measured at a minimum. The number of replicates is depicted by dots of the individual measurements for each experiment in the corresponding figure. To confirm experimental findings generated in the hepatocarcinoma-derived HepG2 cell line, the experimental setting was adjusted and repeated with primary, murine hepatocytes isolated from 5 mice. Furthermore, a rat model was used to reproduce in vitro results in an in vivo context. Those animals had a genetic modification (Wilson animal model) which mimicked in vitro studied conditions. For the different disease states at least 6 rats per group were analyzed. The chelator treatment was performed in 3-4 animals. Additionally, human serum samples of Wilson's disease patients were examined for characteristic, used endpoints. The number of patients per group (normal CP n=8; low CP n=11; diseased n=4) was based on CP levels and additional endpoints. Using these different models, experimental findings could be reproduced.
Randomization	Samples were randomly allocated in this study.
Blinding	The investigators were blinded to group allocation during data collection and analysis.

Reporting for specific materials, systems and methods

We require information from authors about some types of materials, experimental systems and methods used in many studies. Here, indicate whether each material, system or method listed is relevant to your study. If you are not sure if a list item applies to your research, read the appropriate section before selecting a response.

Materials & experimental systems

n/a	Involved in the study	
	x	Antibodies
	x	Eukaryotic cell lines
x		Palaeontology and archaeology
	x	Animals and other organisms
	x	Clinical data
x		Dual use research of concern

Methods

n/a	Involved in the study	
x		ChIP-seq
x		Flow cytometry
x		MRI-based neuroimaging

Antibodies

Antibodies used

rabbit anti-SELENOP (mouse and rat samples; 1:500-1:1000; immunoGlobe, Himmelstadt, Germany; 0122-03), rabbit anti-α1-antitrysin (1:5000; Abcam; 207303), rabbit anti-LRP8 (1:1500 in 5% BSA; Abcam; ab108208), rabbit anti-GAPDH (1:1000; Cell Signaling Technology, Danvers, MA, USA; 2118), rabbit GOLGIN-97 (1:1000; Cell Signaling Technology; 13192), and rabbit anti-ApoE (1:250; Invitrogen; 701241). As secondary antibody, horseradish peroxidase-conjugated goat anti-rabbit IgG (1:50000; Cell Signaling Technology; 7074S) or goat anti-mouse (1:3000; Cell Signaling Technology; 7076S)

Generation of mouse anti-SELENOP according to Hybsier et al., 2017, Redox Biol: Monoclonal antibodies (Ab) were generated essentially as described. In brief, three female BALB/c mice were immunized with an emulsion of purified recombinant SELENOP in TiterMax® Gold adjuvant (Sigma-Aldrich Corp., St. Louis, U.S.A.), followed by a second, third and fourth injection after 30, 60 and 61 days. The protocol had been approved by the local authorities (LAGeSo Berlin, permit No. H 0331/12). The antibody-titer of the mice was determined by an indirect ELISA on day 62 with immobilized recombinant SELENOP and pc rabbit anti-mouse Ab (Z109, Dako Deutschland GmbH, Hamburg, Germany) as detection antibody. The mouse showing the highest anti-SELENOP titer was killed, the spleen removed, lymphocytes isolated, and fused with immortalized murine myeloma cells (PAI, RRID: CVCL_Z070) by PEG1500 (Roche Diagnostics GmbH, Penzberg, Germany).

A macrophage cell suspension was prepared from the abdominal cavity of a mouse as “feeder cells”. Fused hybridoma cells were cultivated first in HAT-DMEM-selection-medium followed by HT-DMEM-medium on a “feeder” cell layer. After ten days, the hybridoma culture supernatant was screened for Ab production, and productive hybridomas with high proliferation grade and Ab titers were expanded, cloned by limiting dilution and subcloned in DMEM medium containing 10% horse serum, 4 mM L-glutamine, 1 µg/mL fungizone, 100 U/mL penicillin, 100 µg/mL streptomycin and 1 mM pyruvate. Eight selected mAb were purified on a ProsepG-column (Merck Millipore, Darmstadt, Germany) and stored at 4 °C in PBS, pH 7.4.

Validation

In human cell culture samples, SELENOP and ApoE antibodies were validated by knockdown experiments resulting in lower intensity of the expected bands. The SELENOP antibody was validated by modulating the selenium supply of cells resulting in almost undetectable signal (see e.g. Fig. 1d).

For the other antibodies, validation data from the manufacturer's homepage were used:
- rabbit anti-α1-antitrysin (1:5000; Abcam; 207303), suitable for Western Blot and reacts with human; in our hands it produced one very specific band at the expected size and accordingly the antibody could also be used for dot blots
- rabbit anti-LRP8 (1:1500 in 5% BSA; Abcam; ab108208), suitable for Western Blot and reacts with human; in our hands the specificity of the antibody was limited but band size matched the expected size and LRP8 was enriched in the membrane/organelle fraction as expected
- rabbit anti-GAPDH (1:1000; Cell Signaling Technology; 2118), suitable for Western Blot and reacts with human; in our hands it produced a specific band of the expected size and showed enrichment in the cytosolic fraction as expected
- rabbit GOLGIN-97 (1:1000; Cell Signaling Technology; 13192), suitable for Western Blot and IF and reacts with human; in our hands it produced a specific band of the expected size and showed enrichment in the membrane/organelle fraction as expected

Eukaryotic cell lines

Policy information about cell lines and Sex and Gender in Research

Cell line source(s)

Cell culture experiments with hepatocarcinoma-derived HepG2 from a male donor (ACC 180 German Collection of Microorganisms and Cell Cultures (DSMZ)) and adenocarcinoma-derived HT-29 cells from a female donor (ACC 299 DSMZ) were performed. The murine hepatocytes were isolated from males.

Authentication

Cells were routinely autheticated by morphological examinations using microscopy.

Mycoplasma contamination

All cell lines were tested negative for mycoplasma on a regular basis.

Commonly misidentified lines
(See ICLAC register)

The cell lines used were not found in the ICLAC register.

Animals and other research organisms

Policy information about studies involving animals; ARRIVE guidelines recommended for reporting animal research, and Sex and Gender in Research

Laboratory animals	Five male C57BL/6JCtrl mice were housed in a 12:12 h light/dark cycle at 22°C and constant humidity at 55%, fed a nutrient-rich standard chow (V1534-300, Ssniff, Soest, Germany) ad libitum and were sacrificed at the age of 20 weeks to isolate primary hepatocytes.<br><br>Samples from male and female LPP rats were used at varying age to induce different stages of disease (control: mean age of 89 weeks; affected group: mean age of 86 weeks; disease onset group: mean age of 94 weeks; diseased group: mean age of 96 weeks). Rats were housed in a 12:12 h light/dark cycle at 22°C and constant humidity at 55% and maintained on an ad libitum Altromin 1314 diet (Altromin Spezialfutter) and tap water. Some rats of the diseased group received the copper chelator methanobactin for 8 days.
Wild animals	The study did not involve wild animals.
Reporting on sex	For the rat experiment, male and female rats were analysed and sex-based analyses have been performed. As there were no or very minor sex differences, we decided to not differentiate according to sex in the final figure.
Field-collected samples	The study did not involve field-collected samples.
Ethics oversight	All animal procedures were approved and conducted following national guidelines. Animal procedures were approved in case of mice by the Ministry of Environment, Health and Consumer Protection of the federal state of Brandenburg, Germany and conducted following national guidelines and institutional guidelines of the German Institute of Human Nutrition Potsdam-Rehbruecke, Germany. Animal experiments with LPP rats were approved by the government authorities of the Regierung von Oberbayern, Munich, Germany, and all animals were treated according to the guidelines for the care and use of laboratory animals of the Helmholtz Center Munich.

Note that full information on the approval of the study protocol must also be provided in the manuscript.

Clinical data

Policy information about clinical studies

All manuscripts should comply with the ICMJEguidelines for publication of clinical research and a completedCONSORT checklist must be included with all submissions.

Clinical trial registration	DRKS00031527
Study protocol	was provided
Data collection	The Wilson's disease patients were recruited between 2011 and 2019 at the University Hospital Heidelberg, Germany as part of the clinical trial 'Biochemical and genetic markers of liver diseases'.
Outcomes	The primary aim of the study is to characterize the patients by recording clinical and laboratory characteristics. Secondary study goals: Follow-up of the patients as part of the medically indicated follow-up controls (approx. every six months to annually) to record the course of the disease.
